# Adipose Tissue Hypoxia Correlates with Adipokine Hypomethylation and Vascular Dysfunction

**DOI:** 10.3390/biomedicines9081034

**Published:** 2021-08-18

**Authors:** Mohamed M. Ali, Chandra Hassan, Mario Masrur, Francesco M. Bianco, Dina Naquiallah, Imaduddin Mirza, Patrice Frederick, Eduardo T. Fernandes, Cristoforo P. Giulianotti, Antonio Gangemi, Shane A. Phillips, Abeer M. Mahmoud

**Affiliations:** 1 Department of Physical Therapy, College of Applied Health Sciences, University of Illinois at Chicago, Chicago, IL 60612, USA; mali37@uic.edu (M.M.A.); shanep@uic.edu (S.A.P.); 2 Integrative Physiology Laboratory, University of Illinois at Chicago, Chicago, IL 60612, USA; 3 Departments of Surgery, College of Medicine, University of Illinois at Chicago, Chicago, IL 60612, USA; chandrar@uic.edu (C.H.); mmasrur@uic.edu (M.M.); biancofm@uic.edu (F.M.B.); pfrede1@uic.edu (P.F.); eduardof@uic.edu (E.T.F.); Giulianotti@uic.edu (C.P.G.); agangemi@uic.edu (A.G.); 4 Division of Endocrinology, Diabetes and Metabolism, Department of Medicine, College of Medicine, University of Illinois at Chicago, Chicago, IL 60612, USA; dnaqui2@uic.edu (D.N.); mmirza24@uic.edu (I.M.)

**Keywords:** visceral adipose tissue, obesity, hypoxia, DNA methylation, TET1, inflammation, adipokines, vascular dysfunction, nitric oxide, flow-induced dilation

## Abstract

Obesity is characterized by the accumulation of dysfunctional adipose tissues, which predisposes to cardiometabolic diseases. Our previous in vitro studies demonstrated a role of hypoxia in inducing adipokine hypomethylation in adipocytes. We sought to examine this mechanism in visceral adipose tissues (VATs) from obese individuals and its correlation with cardiometabolic risk factors. We propose an involvement of the hypoxia-inducible factor, HIF1α, and the DNA hydroxymethylase, TET1. Blood samples and VAT biopsies were obtained from obese and non-obese subjects (*n* = 60 each) having bariatric and elective surgeries, respectively. The analyses of VAT showed lower vascularity, and higher levels of HIF1α and TET1 proteins in the obese subjects than controls. Global hypomethylation and hydroxymethylation were observed in VAT from obese subjects along with promoter hypomethylation of several pro-inflammatory adipokines. TET1 protein was enriched near the promotor of the hypomethylated adipokines. The average levels of adipokine methylation correlated positively with vascularity and arteriolar vasoreactivity and negatively with protein levels of HIF1α and TET1 in corresponding VAT samples, serum and tissue inflammatory markers, and other cardiometabolic risk factors. These findings suggest a role for adipose tissue hypoxia in causing epigenetic alterations, which could explain the increased production of adipocytokines and ultimately, vascular dysfunction in obesity.

## 1. Introduction

Obesity is a considerable public health issue that affects more than one-third (36.5%) of the US population and over 600 million adults worldwide [1]. Obesity is associated with many life-threatening yet preventable comorbidities, such as cardiovascular (CVD), diabetes, and cancer [2]. Therefore, unlocking the complicated interplay between obesity and its comorbidities is critical. Obesity is characterized by a vast accumulation of dysfunctional adipose tissue, and currently, it is largely accepted that these fat depots represent key secretory organs that release multiple bioactive molecules, hormones, and inflammatory cytokines [3]. Obesity is also associated with hypoxia, especially in the expanding adipose tissues, as shown in previous clinical and preclinical studies [4]. It was postulated over a decade ago that adipose tissues could become hypoxic due to obesity-induced adipose accumulation. Several studies in obese mice have shown that severe adiposity can cause hypoxia in adipose tissue, prompting the HIF1α signaling cascade and, as a result, an increase of pro-inflammatory responses [5]. In a previous clinical study, adipose tissue oxygenation measured via a Licox oxygen probe reported lower oxygen levels in obese subjects compared with lean, age-matched controls [6]. In addition, several mechanistic studies proposed that adipose tissue hypoxia could provide molecular mechanisms for chronic inflammation, inflammatory cell infiltration, adipocyte apoptosis, endoplasmic reticulum stress, and mitochondrial dysfunction [7]. In the current study, we hypothesize that hypoxia in the adipose tissues of obese individuals triggers epigenetic changes, mainly in DNA methylation profiles, and subsequently modify gene expression. In this study, we investigated a group of pro-inflammatory adipokines as candidates for the proposed pathway in obese individuals. We suggest that gene expression of these adipokines will be augmented in response to hypoxia and will negatively affect vascular function locally in the adipose tissues (microvessels) and remotely as well (macrovessels).

DNA methylation, in which a methyl group is added to the cytosine residue, is an epigenetic mechanism that regulates the transcription of particular genes. Aberrant DNA hyper- or hypomethylation can result in unscheduled gene silencing or overexpression, respectively. Stimuli in the surrounding microenvironment, such as variations in oxygen levels, can induce aberrant DNA methylation. [8]. Previous studies suggested that hypoxia modifies DNA methylations via interfering with the activity of DNA methyltransferase (DNMT) and the bioavailability of the methyl donor, S-adenosylmethionine (SAM) [9,10]. Lately, DNA hypomethylation was found to be actively induced by Ten-eleven translocation methylcytosine dioxygenases (TETs), of which TET1 is the most significant member [11,12]. TET1 oxidizes 5-methylcytosine (5-mC) to 5-hydroxy-mC (5-hmC); the latter is modified through deamination and decarboxylation that eventually lead to base excision repair and replacement with an unmethylated cytosine [13].

Previous studies in animal models found that knocking out TET1 results in increased global methylation [14]. The role of TET1 has been recently explored in cancer and developmental diseases; however, there is an apparent lack in studying this mechanism in the context of obesity and vascular dysfunction [15]. We previously reported on the role of the HIF1α-inducible TET1 enzyme in causing hypomethylation and inducing mRNA expression of several adipokines in cultured adipocytes [16]. The current study will test this concept in adipose tissue samples obtained from obese and non-obese subjects. This study is likely to fill a knowledge gap by elucidating DNA hypomethylation as an underlying mechanism for the uncontrolled production of inflammatory adipocytokines associated with obesity. Additionally, these findings may point to the HIF1α-TET1 axis as an upstream pathway that could be targeted in future therapeutic investigations. Finally, this study examines the association between these DNA methylation profiles and clinicopathological and physiological measures, including cardiometabolic risk factors and arteriolar flow-induced dilation (FID).

## 2. Materials and Methods

### 2.1. Human Participants

Participants were recruited from the Surgery Clinics at the University of Illinois Medical Center. The obese group consisted of 40 subjects (12 males and 28 women) who underwent laparoscopic gastric sleeve bariatric surgery. The non-obese control group had 40 subjects (18 males and 22 females) who were scheduled for elective surgeries, such as hernia repair. The subjects’ age ranged from 21 to 49 years old, and all the women were premenopausal. For obese subjects, the eligibility criteria included a BMI > 35 kg/m^2^ and the absence of significant chronic diseases or inflammatory conditions that may have confounding effects on the study outcomes. The non-obese controls had to have a BMI < 25 kg/m^2^. In both groups, exclusion criteria included pregnancy, type 1 or 2 diabetes mellitus, cardiac, hepatic, or renal disease, cancer, or acute or chronic inflammatory diseases, such as rheumatoid arthritis. Eligible subjects were educated about the study details in a pre-surgery clinical visit during which blood samples and clinical measurements were obtained. Participants who were interested in participating in the study were given written informed consent. All protocols and procedures of the study followed the standards set by the latest version of the Declaration of Helsinki and were approved by the Institutional Review Board of The University of Illinois at Chicago.

### 2.2. Body Composition and Cardiometabolic Measurements

Physical characteristics, including body weight and BMI, were measured. Total and visceral fat mass and lean mass were assessed using dual X-ray absorptiometry (DXA; iDXA, General Electric Inc). Plasma concentrations of glucose and insulin were quantified in the fasting state as we previously described [17]. These levels were used to calculate the homeostasis model assessment for insulin resistance (HOMA-IR) (fasting insulin (µU/L) × fasting glucose (nmol/L)/22.5 [18]. Lipid profiles, including high-density lipoproteins (HDL), low-density lipoproteins (LDL), total cholesterol, and triglycerides, were measured via enzymatic assays using reagents from Roche Diagnostics (Indianapolis, IN, USA) as we previously described [17]. Hemoglobin A1c (HbA1c) was measured using a kit from Crystal Chem (Elk Grove Village, IL, USA), following the protocol recommended by the manufacturer. Nitric oxide (NO) levels were estimated via measuring its metabolites, nitrate and nitrite, in serum samples using Griess reaction (Cayman Chemicals, Ann Arbor, MI) as we previously published [19]. Briefly, nitrate was converted into nitrite utilizing nitrate reductase; then, the Griess reagents were added, converting nitrite into a dark purple azo compound. Optical density was measured at 540 nm using a multimode plate reader (Molecular Devices, San Jose, CA, USA).

### 2.3. Brachial Artery Flow-Mediated Dilation (FMD)

Ultrasound imaging and brachial artery FMD measurements were performed with Hitachi Prosound Alpha 7 (Hitachi Aloka Medical America, Wallingford, CT, USA) as detailed in our previous publication [17]. The ultrasound probe was placed with a 60° angle tilting 1–3 cm proximal to the antecubital fossa to visualize and measure the diameter of the brachial artery lumen. A blood pressure cuff was placed around the mid-forearm and inflated up to 220 mmHg for 5 min. After cuff deflation, a 300-s-long video was recorded. The imaging protocol involved acquiring 60 s of the baseline (BSL) diameter before inflating the cuff and 300 s for the reactive hyperemia (RH) event induced by the cuff deflation. All images were digitally recorded for post-acquisition analysis using the Automated Edge Detection software system. Relative FMD was calculated using the maximum brachial artery diameter at BSL subtracted from the largest mean values obtained after cuff deflation [%FMD = (RH diameter − BSL diameter / BSL diameter) in mm × 100] as we previously reported [17,20,21,22,23].

### 2.4. Serum Measurements of Inflammatory Biomarkers

We measured C-reactive protein (CRP) concentrations via a high-sensitivity ELISA kit (Crystal Chem, Elk Grove Village, IL, USA) following the manufacturer’s protocol. Briefly, serum samples, standards (0.625–40 ng/mL), and appropriate controls were incubated in the antibody-coated 96-well plate for 60 min followed by a 60-min incubation with the working HRP solution and a 20-min incubation with the substrate solution. Finally, the stop solution was added to terminate the reaction, and the absorbance was measured at 450 nm via a SpectraMax M Series multimode plate reader (Molecular Devices, San Jose, CA, USA). To quantify cytokine levels in plasma, Human High Sensitivity Cytokine A Premixed Magnetic Luminex Performance Assays were performed per the manufacturer’s instructions (R&D). These assays profile 12 analytes (GM-CSF (granulocyte monocyte colony-stimulating factor), IFNγ (interferon-gamma), IL1β (interleukin 1 beta), IL2, IL4, IL5, IL6, IL8, IL10, IL12, TNFα (tumor necrosis factor α), and VEGF (vascular endothelial growth factor)). Samples were incubated with specific antibody-coated magnetic microparticles embedded with fluorophores. After washing steps to remove the non-specific binding, biotinylated antibody cocktails specific to the analytes of interest were added, followed by streptavidin-phycoerythrin conjugate (Streptavidin-PE), which binds to the biotinylated antibodies. Finally, microparticles were resuspended in buffer and read using the LuminexTM MAGPIXTM Instrument System (ThermoFisher Scientific, MA, USA).

### 2.5. Sample Acquisition

On the day of surgery (bariatric or elective surgeries), blood samples were collected before anesthesia, and visceral adipose tissue samples were collected by the surgeon at the beginning of the surgery. Each sample was divided into three equal portions; the first was immediately placed in cold HEPES (2-[4-(2-hydroxyethyl) piperazin-1-yl] ethanesulfonic acid) buffer solution for arteriolar isolation; the second part was fixed in 10% formalin and embedded in a paraffin block; and the third part was snap-frozen in liquid N2 for further molecular analyses.

### 2.6. Adipose Tissue Capillary Density Measurements

Serial 4 µm formalin-fixed, paraffin-embedded (FFPE) cross-sections were obtained and mounted on glass slides. Capillaries were identified using a periodic acid-Schiff stain (Sigma Aldrich, St. Louis, MO, USA) following digestion of glycogen by amylase and hematoxylin counterstain, as we described previously [19]. Images were acquired using a Nikon microscope and analyzed using NIS-Elements Microscope Imaging Software (Nikon, Melville, NY, USA). The number of capillaries per mm^2^ of surface area was determined.

### 2.7. Microvascular Preparation and Flow-Induced Dilation Measurements

Resistance arterioles were isolated from adipose tissue samples and cleaned of excess fat and connective tissues. Isolated arterioles were then prepared for measuring the internal diameter in response to changes in intraluminal pressure gradient as we previously described [19,21,22,23,24]. Briefly, isolated arterioles were cannulated in an organ perfusion chamber using glass micropipettes and a 10-0 nylon Ethilon monofilament suture to secure both ends. Cannulated arterioles were then visualized by an inverted microscope attached to video microscopy. Cannulated arterioles were continuously perfused with heated physiological salt solution (Krebs buffer) consisting of (in mmol/L): KCL (4.4), NaCl (123), CaCl_2_ (2.5), NaHCO_3_ (20), MgSO_4_ (1.2), KH_2_PO_4_ (1.2), and glucose (11). The temperature and pH of the solution were maintained at 37 °C and 7.4, respectively. Krebs buffer was supplied with a mixture of O_2_ (21%), CO_2_ (5%), and N_2_ (74%). The ends of the cannulated arteriole were connected to Krebs-containing reservoirs, and the intraluminal pressure gradient (10–100 cm H_2_O) was created by changing the distance between both reservoirs in equal and opposite directions. Arterioles were preconstructed with angiotensin II (10-6 M) (Sigma Aldrich, St. Louis, MO, USA), and those constricted <30% were excluded from the analysis. The internal arteriolar diameter was measured in response to gradual increases of the intraluminal pressure gradient (10–100 cm H_2_O). Percentage vasodilation was reported as the percentage increase in the arteriolar diameter after each treatment condition relative to the angiotensin II-constricted state. Papaverine (10-4 M) (Sigma Aldrich, St. Louis, MO, USA) was used to measure maximal vasodilation.

### 2.8. Arteriolar Nitric Oxide (NO) Measurements

The generated NO by isolated arterioles was measured as we previously described [19,21,22,23] using the NO Detection Kit (Enzo Life Sciences, Farmingdale, NY, USA). NO measurements were performed in cannulated arterioles in response to a pressure gradient of Δ60 cm H_2_O. Arterioles under flow were incubated with the NO detection reagents followed by excision, washing, and mounting on microscopic slides. Images were taken via fluorescence microscopy (Eclipse TE 2000, Nikon, Japan) at 650 nm. All protocols for incubation, staining, and imaging were fixed in all experiments. Images were then analyzed for fluorescence intensity, and the fluorescent signal was expressed in arbitrary units using Image J software (NIH, Bethesda, MD, USA).

### 2.9. Real-Time PCR (Polymerase Chain Reaction)

Total RNA was isolated from adipose tissue samples using RNeasy mini kits (Qiagen, Germantown, MD). RNA quantity was determined via Qubit Fluorometric Quantification (ThermoFisher Scientific). First, 5 μg RNA were reverse transcribed into cDNA using iScript™ Reverse Transcription Supermix (Biorad Laboratories, Hercules, CA, USA). mRNA expression was determined via real-time RT-PCR using SsoAdvanced Universal SYBR^®^ Green Supermix (Biorad Laboratories), and specific primers for leptin, IL1β, IL6, IL8, IL17, CXCL5, macrophage migration inhibitory factor (MIF), VEGF, TNF-α, and IFNɣ genes were designed using primer3 software v. 0.4.0 and manufactured by Invitrogen Life Technologies (Supplementary Materials Table S1). Gene expression was normalized to GAPDH, and the Livak method (2^−∆∆Ct^) was used to calculate the normalized expression ratio of target genes [25]. All analyses were performed in triplicates.

### 2.10. Global DNA Methylation Analysis

Global levels of DNA methylation and hydroxymethylation were measured via the 5-methyl cytosine (5-mC) DNA and Quest 5-hydroxymethylcytosine (5-hmC) DNA Kits, respectively (Zymo Research, CA, USA). Briefly, DNA was denatured at 98 °C for 5 min, and 100 ng per sample were used to perform the assay per the manufacturer’s protocol. The denatured DNA was incubated at 37 °C in the plate for 1 h, followed by incubation with a mixture of the primary antibody (anti-5-mC or anti-5-hmC) and the secondary antibody. Finally, the horseradish peroxidase (HRP) developer was added to each well. Color development was allowed for 10–60 min, and absorbance was measured at 405–450 nm using a multimode plate reader (M3 Molecular Devices, San Jose, CA, USA). The 5-mC and 5-hmC percentage in each sample was calculated using the absorbance values of the corresponding standard curve. Analyses were performed in triplicates, and the data were represented as the average ± standard error.

### 2.11. Methylation PCR Analysis

DNA methylation analyses were performed using EpiTect Methyl II PCR Array of 94 genes involved in inflammation and autoimmunity. The manufacturer protocol for DNA digestion and PCR analyses was followed. DNA from VAT samples was extracted using a DNeasy Blood & Tissue kit (Qiagen, Chatsworth, CA, USA). DNA concentration and purity were measured using a NanoDrop™ One/OneC Microvolume UV-Vis Spectrophotometer (Thermo Fisher Scientific, Waltham, MA, USA). Only samples with ratios of A260/A230 > 1.7 and A260/A280 > 1.8 were used for the array. DNA was then digested using the EpiTect Methyl II DNA Restriction Kit (Qiagen). A reaction mix was prepared using 2 µg of genomic DNA from each sample mixed with a methylation-sensitive and/or a methylation-dependent restriction enzyme in Restriction Digestion Buffer. Four digestion reactions were set up, M0 (no enzymes), Ms (methylation-sensitive enzyme), Md (methylation-dependent enzyme), and Msd (both enzymes). Enzymes were activated at 37 °C for 6 h followed by heat inactivation at 65 °C for 20 min. The digested DNA was then analyzed using the Human Inflammatory Response and Autoimmunity EpiTect Methyl II Signature PCR Array Profiles (Qiagen) according to the manufacturer’s protocol. The assay is based on detecting the remaining DNA after digestion with methylation-sensitive and methylation-dependent restriction enzymes. The Array profiles the promoter methylation status of a panel of 94 genes, including chemokines, cytokine receptors, and associated proteins, and other inflammatory response and autoimmunity genes in 384 well-plate formats. The real-time PCR assay was performed in a ViiA 7 Real-Time PCR System (Thermo Fisher Scientific). The results were analyzed using the EpiTect Methyl II PCR Array System, which provides an integrated Excel-based template. This template automatically determines gene-specific DNA methylation status via performing all the computations based on the raw threshold cycle (Ct) values using MethylScreenTM technology. This template calculates the percentage of methylated and unmethylated DNA for each gene via normalizing the Ct values of Ms and Md digests with the M0 values.

### 2.12. Western Blotting

RIPA lysis buffer (Cell Signaling) supplemented with protease and phosphatase inhibitor cocktail (MS-SAFE, Sigma-Aldrich) was used to extract total proteins from adipose tissue samples, and a Pierce BCA Protein Assay Kit (Thermo Fisher Scientific) was used to measure the protein concentration. In total, 10 μg of total protein were resolved by 4–12% Bis-Tris gradient gels (Bio-Rad) and transferred to PVDF membranes. These membranes were blocked and incubated with the primary antibodies, HIF1α (H1alpha67) mouse monoclonal antibody, TET1 rabbit polyclonal antibody (Abcam), overnight at 4 °C and then with infrared IRDye^®^-labeled secondary antibodies (LI-COR Biosciences, Lincoln, NE, USA) for 60 min at room temperature. Membranes were washed, dried, and then scanned (800 nm for IRDye800TM antibodies and 700 nm for IRDye680TM antibodies) using an Odyssey Clx infrared imaging system. GAPDH rabbit polyclonal antibody (Cell Signaling) was utilized as a loading control. Acquired images were then quantified using Image Studio (LI-COR) to estimate the intensity of the target protein bands relative to GAPDH in each sample.

### 2.13. Chromatin Immunoprecipitation Assay (ChIP)

Tissues were cut into small pieces (1–3 mm) and DNA-proteins cross-linking was performed via the incubation with 1.5% formaldehyde at 37 °C for 15 min on a rotating platform. Cross-linking was stopped by adding glycine (0.125 M) for 5 min followed by centrifugation, discarding the supernatant, and washing with ice-cold PBS. Tissues were then homogenized in SDS-lysis buffer (50 mM Tris-HCl pH 8.1, 10 mM EDTA, 1% SDS) supplemented with protease inhibitors using a BeadBug Microtube Homogenizer (Benchmark Scientific, Hamilton, NJ, USA). Samples were sonicated on ice for 20 cycles (10 s pulse and 30 s rest) using a Q500 Sonicator (Qsonica, Atkinson, NH, USA). The length of DNA fragments was verified by gel electrophoresis (200 to 800 base pairs). The sonicated chromatin samples were diluted in a ChIP dilution buffer (0.01% SDS, 1.1% Triton X-100, 1.2 mM EDTA, 16.7 mM Tris-HCl, pH 8.1, 167 mM NaCl) (Millipore Sigma, Billerica, MA, USA). The chromatin was then immunoprecipitated via Dynabeads coated with normal rabbit IgG or TET1 antibody overnight at 4 °C. Following elution, the protein–DNA cross-link was reversed by heating at 65 °C for 6 h. The immunoprecipitation of TET1 was verified by Western blotting. The eluted DNA was purified via MiniElute PCR purification Kit (Qiagen) followed by quantification of the target adipokine promoters by real-time PCR assays as described above. Primers for the promoters of 28 adipokines were designed to produce an amplicon that encompass the region −500 to 100 relative to the transcription start site using the Eukaryotic Promoter Database (EPD) and primer3 online program (Supplementary Materials Table S2).

### 2.14. Statistical Analyses

Data were shown as average ± standard error (SE), and statistical significance was achieved if *p* < 0.05. For the between-group comparison, a non-paired Student’s test was used. When there were more than two comparisons, one-way ANOVA followed by an appropriate post hoc test was used. A total methylation score was calculated by averaging the methylation percentages of all differentially methylated inflammatory genes. The statistically significant linear relationship between continuous variables was tested using a bivariate Pearson correlation. Multiple regression analysis was used to explore the independent association between VAT hypoxia and average adipokine promoter methylation. In this model, we included VAT % and capillary density, HOMA-IR, blood pressure, plasma NO and IL6, arteriolar FID at Δ60, and VAT levels of HIF1α and TET1. Analyses were performed using SPSS statistical package version 28 (SPSS Inc, Chicago, IL, USA). Metascape (http://metascape.org/gp/index.html#/main/step1 was accessed on 22 July 2021) and used for the pathway enrichment analysis. First, all statistically enriched terms (GO/KEGG terms, canonical pathways, hall mark gene sets, etc.) were identified, and accumulative hypergeometric *p*-values and enrichment factors were calculated and used for filtering. The remaining significant terms were then hierarchically clustered into a tree based on Kappa-statistical similarities among their gene memberships. Finally, a 0.3 kappa score was applied as the threshold to cast the tree into term clusters

## 3. Results

### 3.1. Cardiometabolic Risk Factors

The subject characteristics, including body weight, body mass index (BMI), and waist circumference, are summarized in Table 1. Total body fat and VAT percentages measured via DEXA scanning were significantly higher in obese subjects than non-obese controls (*p* < 0.0001). Cardiometabolic risk factors, including heart rate, systolic and diastolic blood pressure, glucose metabolism, and lipid profile, are also displayed in Table 1. Heart rate and diastolic and systolic blood pressure were significantly higher in the obese group. The average fasting blood glucose (FPG) and glycosylated hemoglobin (HbAlc) levels were not statistically different between the two groups. Still, the average fasting plasma insulin (FPI) and the insulin resistance index, HOMA-IR, were significantly lower in the control group compared to obese subjects. Only HDL showed a statistically significant difference in the lipid profile, being 25% higher (*p* < 0.001) in non-obese controls compared to obese subjects. C-reactive protein (CRP) was 4.7-fold higher in obese subjects compared to the controls, while serum nitric oxide (NO) was 41% higher in the latter group. Vitamin D levels in the obese subjects were also lower than in the controls.

Folate and vitamin B12 levels in the blood have previously been linked to the efficiency of one-carbon metabolism and, consequently, DNA methylation status [17,26]. Our data showed higher plasma concentrations of folate and B12 in controls compared to obese subjects. Homocysteine (Hcy) is a byproduct during the one-carbon metabolism cycle, a critical step in the DNA methylation process. Increased plasma levels of Hcy have been identified when nutritional factors that function as methyl donors, such as folate and vitamin B12, are deficient [17]. Hyperhomocysteinemia is a well-established, independent risk factor for cardiovascular disease (CVD) [27,28]. According to the findings of this study, obese participants had a ~30% rise in plasma Hcy relative to controls. Alcohol intake was also assessed using a questionnaire, as it has been shown to impair folate and B12 absorption and bioavailability and raise Hcy levels in the blood [29]. Alcohol consumption was found to be higher in the obese (63%) compared to the non-obese group (37%); most of this difference was in the moderate (obese: 30%, control: 17%) and heavy (obese: 18%, control: 0%) drinking categories (Table 2).

### 3.2. Analysis of VAT and Circulating Cytokines

**Figure 1:**
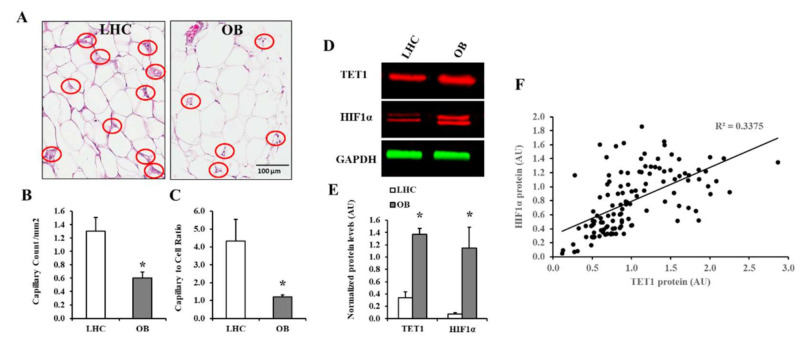
Adipose tissue’s vascularity and protein levels of HIF1α and TET1. (**A**) Representative photomicrographs of adipose tissue biopsies stained with an amylase-periodic acid Schiff (PAS) histochemical stain for capillary detection. Capillaries were indicated by red circles. (**B**,**C**) Graphical representations of capillary count per mm^2^ and capillary to cell ratio in VAT samples obtained from obese and non-obese subjects (*n* = 60, each). (**D**,**E**) Western blot analysis and quantification of the normalized signal intensity of HIF1α and TET1 proteins in VAT samples obtained from obese subjects and controls (*n* = 60, each). (**F**) A scatter plot and Spearman correlation coefficient (r) of HIF1α and TET1 proteins in VAT. Results are shown as means ± standard error (SE), and the * symbol reflects a *p*-value < 0.05 when comparing obese subjects with controls.

In VAT, the average number of capillaries per square millimeter of surface area (capillary density and mean capillary to cell ratio were lower in obese subjects than non-obese control (Figure 1A,B). Capillary density was found to be positively correlated with plasma NO (*r* = 0.65, *p* = 0.001) and negatively correlated with BMI (*r* = −0.81, *p* < 0.0001), total fat (*r* = −0.76, *p* < 0.0001), VAT mass (*r* = −0.91, *p* < 0.0001), fasting plasma insulin (*r* = −0.45, *p* = 0.006), HOMA-IR (*r* = −0.68, *p* = 0.001), and C-reactive protein (*r* = −0.63, *p* = 0.01). VAT of obese subjects and non-obese controls was probed for HIF1α and TET1 via the Western blotting technique (Figure 1C). The protein level of HIF1α was 14-fold higher in obese subjects than controls (*p* < 0.01).

HIF1α protein concentrations in VAT correlated positively with BMI, total and visceral fat mass, fasting plasma insulin and Hcy levels, and alcohol consumption. HIF1α correlated negatively with lean mass, HDL, plasma NO, folate, vitamin B12, and vitamin D. These associations are summarized in Table 3. Obese subjects also showed increased TET1 protein expression compared to the non-obese group (3-fold *p* < 0.001). TET1 protein levels in VAT showed a significant positive correlation with HIF1α protein levels, as shown in Figure 1D,E (*p* < 0.001). TET1 and HIF1α proteins correlated significantly (Figure 1F). They both had the same relationships with anthropometric and cardiometabolic risk variables (Table 3).

**Figure 2:**
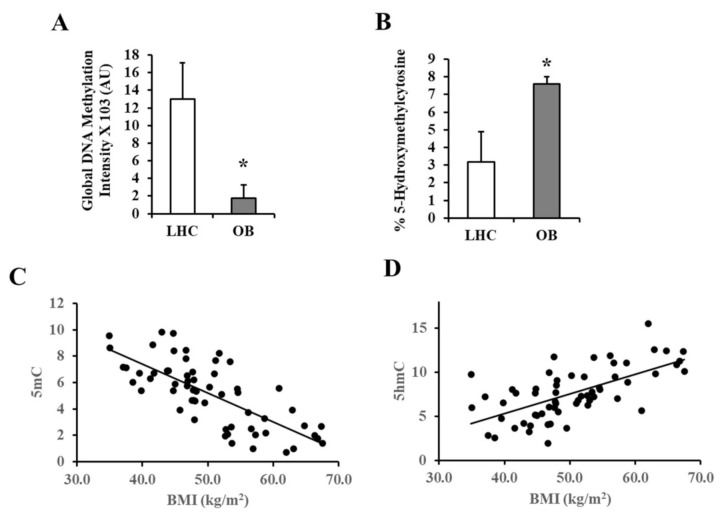
Global methylation and hydroxymethylation in VAT-derived DNA. (**A**,**B**) Representative charts for the quantification of 5-mC and 5-hmC in VAT-derived DNA samples obtained from obese and non-obese subjects (*n* = 60, each). (**C**,**D**) Scatter plots and Spearman correlation coefficient (r) of 5-mC and 5-hmC measurements and BMI. Results are shown as means ± standard error (SE), and the * symbol reflects a *p*-value < 0.05 when comparing obese subjects with controls.

Global DNA methylation and hydroxymethylation were measured in VAT using the 5-methylcytosine (5-mC) DNA and Quest 5-hydroxymethylcytosine (5-hmC) DNA Kits, respectively. Control subjects had 6.5-fold higher global levels of methylated DNA than obese subjects (Figure 2A). The obese group, on the other hand, had 1.1-fold higher global levels of hydroxymethylated DNA (Figure 2B). Figure 2C,D indicate that methylated DNA correlated negatively with BMI (*r* =−0.74, *p* < 0.001) while hydroxymethylated DNA correlated positively with BMI (*r* = 0.64, *p* < 0.001).

**Figure 3:**
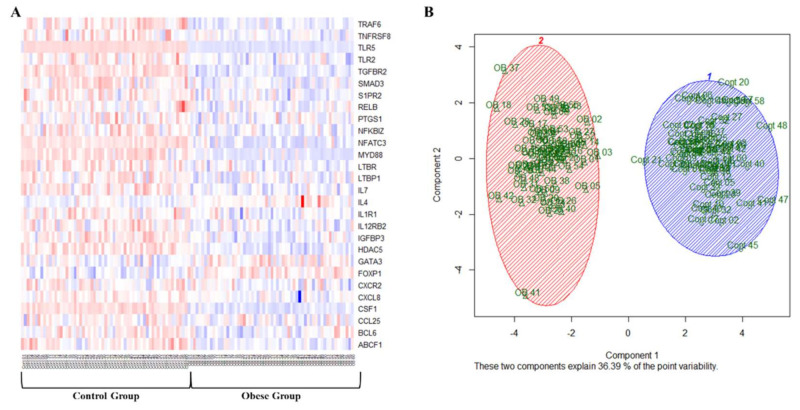
Differentially methylated adipokines in VAT biopsies. (**A**) A representative heatmap of the promoter methylation % of 24 differentially methylated adipokines in VAT samples obtained from obese and non-obese subjects (*n* = 60, each). The heatmap uses a color key that ranges from red (high) to blue (low) based on methylation level. (**B**) A scatterplot of the first two principal components demonstrates a general separation of the obese and non-obese phenotypes.

Gene-specific methylation was assessed in VAT using an array that comprises 94 genes known to be involved in inflammation and immune function. The average percentage of methylated and unmethylated promoters in all genes pooled together reflected higher methylation in the controls (60%) compared to obese subjects (42%, *p* < 0.0001). Approximately 77% of the genes in the array displayed lower levels of promoter methylation in obese subjects relative to controls, with pro-inflammatory genes being the most affected. After correction for multiple comparisons test analyses, 28 out of the 94 genes in the array demonstrated a significant difference in promoter methylation between obese and control subjects, as can be clearly seen in the heat map in Figure 3A. A scatterplot of the first two principal components that captured ~36% of the data variance demonstrates a general separation of the obese and non-obese phenotypes. These findings indicate that the methylation profiles of these inflammatory genes were able to distinguish between non-obese and obese subjects and that the differences were significant and informative enough to separate the two groups (Figure 3B). Table 4 summarizes the percentage of methylated promoters in these genes in both the control and obese groups.

The methylation percentage in the promoters of these 28 genes was found to be significantly associated with many anthropometric and cardiometabolic risk factors. To summarize these associations, we calculated a total methylation score by averaging the methylation percentages of all differentially methylated inflammatory genes. The calculated methylation score correlated negatively with BMI, total fat and VAT percentage, blood pressure, plasma insulin and Hcy, alcohol consumption, VAT vascularity, TET1 and HIF1α protein levels, and circulating inflammatory markers. Positive correlations were found between the methylation score and variables, such as HDL, plasma NO, vitamin B12, folate, vitamin D, and total lean %. Pearson correlation (*r*) and *p* values for the association between gene promoter methylation and cardiometabolic risk measurements are summarized in Table 5. A multiple regression was run to predict adipokine methylation from VAT % and capillary density, HOMA-IR, blood pressure, plasma NO and IL6, arteriolar FID at Δ60, and VAT levels of HIF1α and TET1. These variables statistically significantly predicted the adipokine methylation average, F (9, 110) = 31.948, *p* < 0.001, R^2^ = 0.941. All variables added statistically significantly to the prediction, *p* < 0.05. VAT levels of HIF1α (standardized coefficient β = −0.515, *p* = 0.001) and arteriolar FID (standardized coefficient β = 0.81, *p* < 0.001) were significantly associated with the dependent variable, the methylation score, after adjustment for other independent variables in this model.

**Figure 4:**
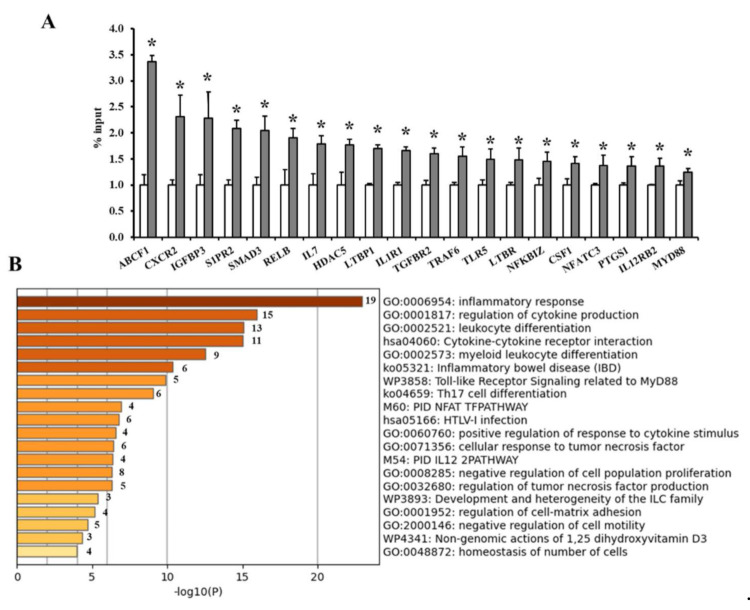
TET1 occupancy at adipokine promoters and gene enrichment analysis. (**A**) Average binding occupancy of TET1 across adipokine promoters in obese subjects measured via ChIP-qPCR assays and normalized to non-obese controls (*n* = 60, each). Results are shown as means ± standard error (SE), and the * symbol reflects a *p*-value < 0.05 when comparing obese subjects with controls. (**B**) Heatmap of enriched terms across the input differently methylated gene lists, colored by *p*-values, via the Metascape.

To corroborate the enrichment of TET1 in the promoters of the differentially methylated adipokines, ChIP-qPCR experiments were performed using an anti-TET1 antibody and primers designed to target the adipokine promoters. We observed a significant enrichment of TET1 in the promoters of 20 adipokines, including ABCF1 (3.4-fold), CXCR2 and IGFBP3 (2.3-fold), S1PR and SMAD3 (2-fold), and IL7 and HDAC5 (1.7-fold). Figure 4A summarizes the fold enrichments of TET1 in the adipokine promoters in VAT from obese subjects normalized to the non-obese controls. The functional enrichment analysis with Metascape found that the differentially methylated genes between obese and non-obese VAT samples were significantly enriched in the pro-inflammatory response, cytokine production and interaction, and leucocyte differentiation (*p* < 0.05, Figure 4B).

**Figure 5:**
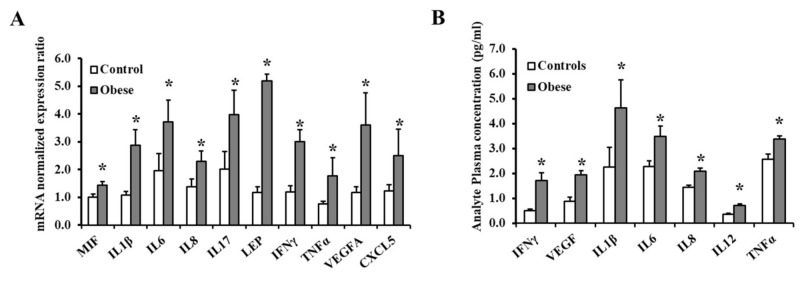
The expression of differentially methylated genes in VAT and circulation. (**A**) Quantitative assessment of the mRNA levels of 10 differentially expressed adipokines in VAT samples (60 obese and 60 controls) using real-time PCR. (**B**) Circulating concentrations of inflammatory cytokines measured in obese subjects (*n* = 60) and non-obese controls (*n* = 60) using specific ELISA assays. Results are shown as means ± standard error (SE), and the * symbol reflects a *p*-value < 0.05 when comparing obese subjects with controls.

To test whether the different methylation profiles of these adipokines had a functional effect on gene transcription, we measured the mRNA expression of the differentially methylated genes via qPCR. Our results showed significant increases in mRNA levels of 10 out of the 28 pro-inflammatory genes with pronounced hypomethylation in obese subjects compared to the non-obese controls (Figure 5A). On the top of these genes are LEP, VEGF, IL1β, and IFNγ (*p* < 10^−10^). Despite significantly decreased promoter methylation in the obese population, there were no statistically significant variations in mRNA expression between obese and non-obese subjects for the remaining 18 genes. Circulating levels of inflammatory cytokine were measured using Human High Sensitivity Cytokine Magnetic Luminex Performance Assays (R&D). As outlined in the methods section, this assay consists of 12 analytes. Seven of these analytes (IFNγ, IL1β, IL6, IL8, IL12, TNFα, and VEGF) were found to be significantly higher in obese subjects than in controls (Figure 5B).

### 3.3. Vascular Measurements

**Figure 6:**
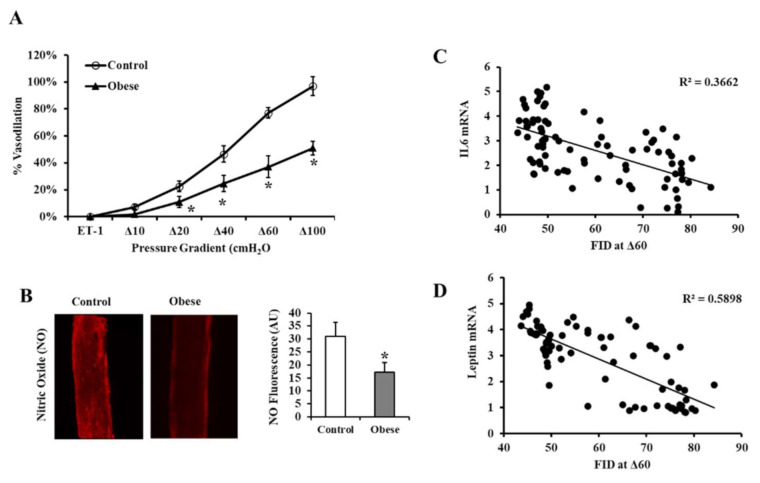
FID measurements in VAT-isolated resistance arterioles. (**A**) FID measurements correspond to increasing intraluminal pressure gradients of 10–100 cmH_2_O in VAT arterioles obtained from obese (*n* = 60) and non-obese (*n* = 60) subjects. (**B**) Representative images by fluorescence microscopy of NO generation in VAT-isolated arterioles (*n* = 60, each). (**C**) Charts represent NO fluorescent signals measured and expressed in arbitrary units using NIH Image J software. (**D**) Scatter plots and Spearman correlation coefficient (r) of leptin and IL6 mRNA levels and arteriolar FID. Results are shown as means ± standard error (SE), and the * symbol reflects a *p*-value < 0.05 when comparing obese subjects with controls.

The FID measured in isolated VAT arterioles in response to increasing the intraluminal pressure gradient (Δ10–Δ100 cmH_2_O) was higher in non-obese than obese subjects across all pressure gradients (Figure 6A). FID at Δ60 cmH_2_O, representing the mean physiological arteriolar pressure within the human body, was 52% lower in obese subjects than in controls. In addition to FID, the flow-induced NO production was measured in the isolated VAT arterioles using a fluorescent NO indicator (Figure 6B). The signal intensity was measured using the NIH Image J software. Figure 6B indicates that the obese group’s NO generation was 45% lower than the non-obese controls (Figure 6C). The FID of the isolated VAT arterioles correlated negatively with the mRNA expression of the differentially expressed inflammatory cytokines measured in the corresponding VAT biopsies, with IL6 (Figure 6C) and leptin topping the list (Figure 6D). Arteriolar FID also correlated with several cardiometabolic risk factors, VAT levels of HIF1α and TET1, circulating markers of inflammation, and a lower adipokine methylation score in VAT (Table 6).

**Figure 7:**
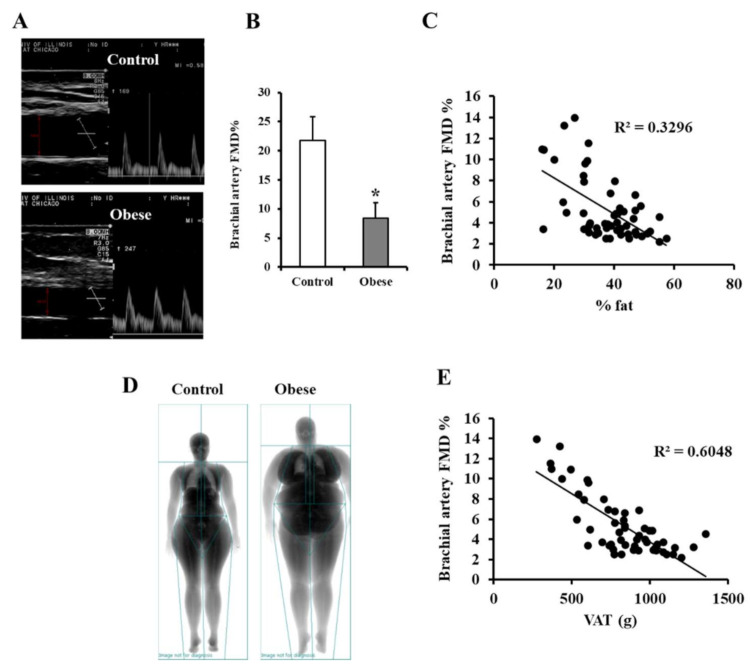
FMD measurements in the brachial artery. (**A**) Duplex B-mode/PWD (pulsed wave Doppler) ultrasound of brachial artery FMD showing a long axis scan of the artery and a pulsed wave Doppler blood velocity profile. (**B**) By subtracting the average baseline diameter from the largest mean values obtained following cuff deflation, the percentage of brachial artery FMD was estimated in obese subjects (*n* = 60) and non-obese controls (*n* = 60). (**C**) A scatter plot and Spearman correlation coefficient (r) of FMD and total fat % measured via DEXA scanning. (**D**) Representative images of total body composition measurements by dual-energy X-ray absorptiometry (DEXA) scanning in obese and control subjects. (**E**) A scatter plot and Spearman correlation coefficient (r) of FMD and total VAT % measured via DEXA scanning. Results are presented as means ± SE, and the * symbol reflects a *p*-value < 0.05 when comparing obese subjects with controls.

Pulsed wave Doppler ultrasound was used for measuring brachial artery FMD (Figure 7A). The baseline diameter of the brachial artery was not statistically different between the two groups, yet the %FMD in response to reactive hyperemia was 1.6-fold lower in obese subjects than non-obese controls (*p* < 0.001; Figure 7B). While FMD was negatively correlated with total body fat percentage (Figure 7C), the correlation with VAT mass estimated via DEXA scan was more statistically significant (Figure 7D,E), highlighting the contribution of VAT’s volume to vascular dysfunction in obese subjects. Brachial artery FMD, like arteriolar FID, was associated with multiple cardiometabolic risk factors and a lower adipokine methylation score, as shown in Table 6.

## 4. Discussion

The interaction between the body and various external factors, such as nutrition, physical activity, and other lifestyle factors [30], and internal ones, such as the hormonal and secretory milieu in the body, is reflected upon epigenetic profiles [31]. As a result, epigenetic alterations in pathologic circumstances like obesity and how they contribute to cardiovascular and metabolic morbidity receive more attention. Numerous epigenetic alterations have been linked to obesity [32]; nevertheless, the clinical consequences and links between these patterns and specific metabolic or vascular profiles in obese people need to be investigated further. The majority of published epigenetic patterns were evaluated in easily accessible samples, such as peripheral blood cells, with a lack of equivalent findings in metabolically relevant tissues, such as the adipose tissue. Furthermore, research into the mechanistic basis of these epigenetic patterns is warranted. To this purpose, we looked at the methylation patterns of several inflammation-related genes in the adipose tissues of obese and non-obese individuals, as well as their relationship with cardiometabolic risk factors and vascular function. We also analyzed the HIF1α and TET1 proteins and their association with the DNA methylation patterns measured in this cohort.

VAT has been more inflammatory than subcutaneous adipose tissues and is linked to high levels of systemic inflammation [33]. Our findings showed that pro-inflammatory gene promoters in VAT-isolated DNA were hypomethylated in obese participants compared to non-obese controls. Reduced promoter methylation was statistically significant in 28 genes and was linked to TET1 enrichment in the promoter of 20 these adipokines and higher mRNA expression in 10 of them, including LEP, VEGF, IL1β, and IFNΥ. Previous studies have suggested a link between hypoxia and inflammation [34]; the latter is a defining feature of obesity and is regarded as the underlying mechanism of various obesity-related metabolic and cardiovascular comorbidities [35,36]. As the adipose tissue bulk increases, hypoxia develops, contributing to the progression of inflammation in adipocytes and adjacent tissues [37]. In the current research, we examined the association between adipokine promoter hypomethylation and hypoxia defined by reduced vascularity and elevated HIF1α in VAT.

The role of hypoxia in generating inflammation in adipose tissues has been reported in animal models and proposed to be partially dependent on HIF1α [38,39]. However, the intermediate mechanisms linking hypoxia and inflammation in adipose tissues remain to be characterized. In this study, we suggest an epigenetic mechanism to modulate the effect of the dysregulated HIF1α pathway on adipose tissue inflammation. This mechanism entails the TET1 protein actively demethylating DNA, resulting in DNA hypomethylation. We propose that inflammatory adipokines are downstream targets for this mechanism, leading to enhanced adipokine expression. High levels of hypoxia were shown to induce TET1 expression, 5-hmC accumulation, and global DNA hypomethylation in hepatoblastoma cells [40], breast cancer cells [41], and other malignancies [42,43]. Despite the growing body of evidence suggesting TET enzymes play a role in hypoxia-induced hypomethylation, this involvement has only been documented in the setting of cancer, and equivalent findings have not been established in adipose tissues, which are highly likely to be hypoxic in obese people.

The current study’s findings indicate a direct association between VAT hypoxia and elevated TET1 protein and adipokine hypomethylation levels, implying that TET1 plays a role in the regulation of hypoxia-induced inflammation. This role was demonstrated mechanistically in our prior investigations, which showed an increase in HIF1α and TET1 protein levels and decreased global and gene-specific DNA methylation in adipocytes cultured under hypoxic conditions [16]. Together, our previous mechanistic findings in adipocytes along with the current correlation observed between HIF1α and TET1 in the adipose tissue suggest that obesity-related production of inflammatory cytokines could be mediated by an epigenetic process that is sensitive to changes in the adipose tissue microenvironment, specifically oxygen levels.

It is now widely understood that white adipose tissues secrete a significant portion of circulating adipocytokines, linking obesity to systemic inflammation and related comorbidities. In the current study, the percentage of promoter methylation in differentially methylated genes (used to calculate the methylation score) was strongly associated with body composition, cardiometabolic risk factors, and vascular function. Specifically, lower methylation scores were associated with higher body mass, waist circumference, total and visceral fat percentage, heart rate, blood pressure, and fasting plasma insulin. Despite the lack of significant changes in glucose levels, insulin levels in the obese group were significantly higher, as were measures of insulin resistance (HOMA-IR). This trend implies insulin resistance in the obese population, which is frequently associated with inflammation [44]. We also observed an adverse association between the methylation score and circulating inflammatory cytokines, such as IFN, IL1, IL6, and TNF, suggesting that adipose tissues contribute at least in part to the circulating cytokines. Reduced adipokine methylation levels were shown to be directly associated with vitamin D deficiency and lower NO bioavailability observed in obese patients. These findings are consistent with previous association studies showing a correlation between low vitamin D levels to systemic inflammation and increased risk of CVD in obese individuals [45]. However, further evidence is required to support this association at the physiological and molecular levels given the lack of comparable findings in controlled clinical trials.

Several differentially methylated CpG sites have been associated with obesity in epigenome-wide association studies (EWASs). Some of these studies found that the methylation of CpGs in genes involved in lipid metabolism and inflammatory pathways was altered in obese people compared to lean healthy controls and that these differentially methylated CpG sites could predict future morbidities, such as type 2 diabetes, in the participants [46,47,48]. However, these findings were not consistent across EWASs [49,50,51], which could be attributable to the increased risk of false-positive results in EWASs. On the other hand, studies that targeted candidate genes and specific pathways reported consistent associations between obesity and abnormal methylation patterns of genes related to adipogenesis, insulin resistance, and lipid metabolism, such as PPARG (peroxisome proliferator-activated receptor-gamma) [52], RXRA (retinoid X receptor-alpha methylation), leptin [53], adiponectin [54], and IGFBP-2 (insulin-like growth factor binding protein 2) [55].

Another potential source of heterogeneity across EWASs could be the use of DNA from whole blood, which contains diverse cell groups with varied epigenetic profiles. Therefore, it is critical to conduct further research to consider tissue-specific individual genes and detailed pathways that could be candidates for novel therapeutic and preventive interventions in obesity. To this end, the current study identified 28 adipokines with hypomethylated promoters in VAT from obese individuals compared to controls. Interestingly, in our previously published arrays using blood samples, 6 of these 28 genes (ABCF1, CCL25, CSF1, IL7, MYD88, TLR5) had similar patterns of promoter hypomethylation in obese subjects compared to controls. Together, these findings imply a degree of concordance between adipokine methylation patterns in blood and adipose tissues. However, larger gene arrays will be required in the future to assess the similarity in adipokine methylation profiles between blood and adipose tissue samples.

Epidemiological studies have linked global DNA hypomethylation to an elevated risk of coronary heart diseases [56,57] and stroke [57,58]. Nonetheless, clinical investigations examining the relationship between DNA methylation patterns and vascular measures are sparse. The current study’s findings reveal a significant relationship between adipokine hypomethylation and impaired vascular function, including macrovascular (brachial artery FMD) and microvascular (arteriolar FID) function as well as systemic and arteriolar NO generation. To the best of our knowledge, this is the first study to correlate these vascular characteristics to VAT-specific DNA methylation in obese and non-obese adults.

The fact that CVD is one of the most common preventable causes of death in obese people gives tremendous weight to the current findings and motivates future research into the epigenetic basis of obesity-related vascular dysfunction. Collectively, our results suggest that DNA methylation in VAT might be employed as an epigenetic marker of inflammation and impaired vascular function in obese individuals. These findings further support our assumption that epigenetic modifications could serve as therapeutic targets in obesity and related metabolic disorders. More research with a bigger sample size is needed to back up this claim.

Obese people have a relatively high frequency of micronutrient deficiencies, despite their high-calorie intake [59]. This phenomenon has been explained by obese people’s propensity to choose high-calorie, nutritionally deficient diets or by the fact that excess adiposity alters the storage and availability of micronutrients. Some of these micronutrients, such as vitamin B, function as cofactors in critical metabolic pathways in the body, such as the folate cycle [26]. Through a sequence of enzymatic reactions cofactored by vitamins B6 and B12, dietary folate is transformed to dihydrofolate, then to tetrahydrofolate (THF), and subsequently converted to 5-methyl THF. The latter donates its methyl group to Hcy forming methionine, which is required to produce the ultimate methyl donor, S-Adenosylmethionine [26]. As a result, we set out to investigate the status of folate, vitamin B12, and Hcy in obese people and their relationship with DNA hypomethylation. Indeed, when compared to controls, obese people had lower plasma levels of folate and vitamin B12 and more significant amounts of Hcy. This pattern indicates a dysfunctional folate cycle, which is likely to impact the methylation process negatively.

In the current study, levels of folate and vitamin B12 correlated positively with adipokine methylation, lending support to this theory. These findings imply that methyl donor insufficiency may play a role in DNA hypomethylation in obesity, corroborated by prior studies that found reduced folate and vitamin B12 concentrations in overweight and obese adults. We also evaluated alcohol consumption in this study as it has been shown to interfere with folate absorption. Alcohol intake was higher in the obese group than controls, and it was negatively associated with adipokine methylation levels. The link between methyl donor insufficiency and DNA hypomethylation has previously been identified in cancer [60,61] and neural tube defects [62]. Still, there has been little research into this link in the setting of mild chronic inflammation observed in obesity and cardiometabolic diseases. Therefore, future research into the effect of methyl donor supplementation on global and gene-specific methylation, particularly in obesity, is necessary. DNA methylation is a reversible system that responds to changes in various internal and external environmental variables. Because of this plasticity, DNA methylation should be a target for multiple preventive and therapeutic interventions aiming at restoring healthier methylation patterns and vascular function in obese individuals.

The current study has several strengths to be noted. First, a diverse set of inflammation-related genes were analyzed in surgically obtained VAT from morbidly obese and non-obese subjects using validated promoter-targeting enzyme restriction assays. In addition, the expression of the differentially methylated genes was evaluated to test the functional implications of these methylation patterns. Furthermore, DNA methylation data were examined for their association with crucial cardiometabolic risk variables, methyl-donor nutrient status (folate and vitamin B12), alcohol use, and in vivo and ex vivo vascular measures. Finally, unlike prior studies that exclusively examined vascular function in large conduit arteries like the brachial and femoral, we examined FID in small resistance arterioles, which are likely to be immediately affected by VAT molecular alterations. Moreover, small arterioles are the primary site of peripheral resistance and blood pressure regulation, making this research clinically relevant and highly impactful. However, there are certain limitations to this study. First, our sample size was modest, limiting our statistical power to identify significant changes in several genes after accounting for multiple testing. Furthermore, the small sample size may have reduced the strength of the association between DNA methylation and several of the cardiometabolic risk variables evaluated. Second, while data on alcohol consumption was gathered, detailed information on nutrition or diet was not obtained or adjusted for in the analyses. Lastly, the current study is cross-sectional in nature. As a result, drawing causal inferences or recommending a specific direction for the relationship between obesity and DNA methylation is improper. Therefore, future longitudinal studies are required to understand further the nature of this association and the impact of treatment interventions on DNA methylation profiles.

## 5. Conclusions

In summary, this study identified DNA methylation as a novel outcome for adipose tissue hypoxia and adipokines as downstream targets for this mechanism. Furthermore, the current study discovered a significant correlation between adipokine hypomethylation and impaired FID of VAT-embedded resistance arterioles. Finally, adipokine methylation was found to be significantly related to the blood levels of methyl donors, alcohol intake, inflammatory markers, and various cardiometabolic risk factors. Collectively, the outcomes of this study reveal a relationship between adipokine hypomethylation and vascular dysfunction in obese people.

## Figures and Tables

**Table 1:** Subject characteristics and cardiometabolic risk factors.

Variable	Non-Obese Controls	Obese Bariatric Patients	*p*-Value
n	60 (18 ♂)	60 (12 ♁)	
Age, y	35.8 ± 1.8	36.4 ± 1.8	0.541
Weight, kg	75.8 ± 2.2	143.2 ± 3.4 *	<0.0001
BMI, kg/m^2^	24.9 ± 0.6	51.1 ± 1.3 *	<0.0001
Waist circumference, cm	89.2 ± 1.9	133.5 ± 3.8 *	<0.0001
Body fat, %	32.2 ± 2.5	52.5 ± 1.1 *	<0.0001
Body lean, %	65.3 ± 2.4	46.8 ± 1.2 *	<0.0001
VAT mass, kg	0.7 ± 0.1	2.2 ± 0.2 *	<0.0001
Heart rate, bpm	75 ± 4	86 ± 3 *	0.001
Systolic BP, mmHg	118 ± 2	135 ± 6 *	0.005
Diastolic BP, mmHg	74 ± 1	82 ± 3 *	0.008
FPG, mg/dL	92 ± 1	99 ± 6	0.211
FPI, µU/mL	8.3 ± 0.3	16.1 ± 2.2 *	<0.001
HOMA-IR	1.9 ± 0.1	5.2 ± 0.5 *	<0.001
HbA1c, %	5.4 ± 0.2	5.8 ± 0.2	0.148
Total cholesterol, mg/dL	156 ± 10	165 ± 10	0.327
HDL, mg/dL	56 ± 6	45 ± 2 *	<0.001
LDL, mg/dL	87 ± 8	96 ± 6	0.221
Triglycerides, mg/dL	92 ± 11	110 ± 7	0.160
C-reactive protein, mg/dL	0.7 ± 0.1	4.0 ± 0.4 *	<0.0001
Plasma NO, µmol/L	4.5 ± 0.6	3.2 ± 0.4 *	0.002
Folate, ng/mL	18.7 ± 0.7	14.8 ± 0.8 *	0.0004
Vitamin B12, pg/mL	562 ± 18	398 ± 26 *	<0.0001
Homocysteine, µmol/L	8.7 ± 0.2	11.4 ± 0.3 *	0.003
Vitamin D, ng/mL	37.2 ± 4.7	16.9 ± 1.1 *	<0.0001

*p*-values are from non-paired Student’s test (* *p* < 0.05). Abbreviations: BMI, body mass index; VAT, visceral adipose tissues; BP, blood pressure; FPG, fasting plasma glucose; FPI, fasting plasma insulin; HOMA-IR, homeostatic model assessment for insulin resistance; HbA1c, glycosylated hemoglobin; HDL, high-density lipoprotein; LDL, low-density lipoprotein; NO, nitric oxide.

**Table 2:** Alcohol consumption categories in obese subjects and non-obese controls.

Alcohol Consumption Category	Non-Obese (*n* = 60)	Obese (*n* = 60)	*p*-Value
Abstainers	38 (63%)	22 (37%)	0.002
Light drinkers	12 (20%)	9 (15%)	
Moderate drinkers	10 (17%)	18 (30%)	
Heavy drinkers	0 (0%)	11 (18%)	

*p*-values are from χ^2^ tests. Categories: Light drinkers, less than one time/month with less than five drinks/time, 1–3 times/month with less than three drinks/time, or 1–2 times/week with less than two drinks/time. Moderate drinkers, 1–3 times/month with 3–4 drinks/time, 1–2 times/week with 2–4 drinks/time, or 3–6 times/week with less than 2 drinks/time. Heavy drinkers, any quantity and/or frequency that is more than moderate drinkers.

**Table 3:** Correlations between HIF1α and TET1 protein levels and cardiometabolic risk factors.

	HIF1α Protein in VAT		TET1 Protein in VAT	
	Pearson Correlation	Sig (2-Tailed)	Pearson Correlation	Sig (2-Tailed)
Body weight	0.925 **	7.5 × 10^−37^	0.884 **	1.9 × 10^−29^
Waist circumference	0.767 **	6.1 × 10^−9^	0.748 **	2.2 × 10^−8^
BMI	0.960 **	5.9 × 10^−48^	0.929 **	6.1 × 10^−38^
Total fat %	0.782 **	7.3 × 10^−11^	0.763 **	3.6 × 10^−10^
Total lean %	−0.775 **	1.3 × 10^−10^	−0.755 **	6.7 × 10^−10^
VAT mass	0.620 **	2.1 × 10^−4^	0.0591 **	4.6 × 10^−4^
Heart rate	0.157	7.5 × 10^−2^	0.186 *	4.4 × 10^−2^
Systolic BP	0.393 **	9.9 × 10^−5^	0.383 **	1.5 × 10^−4^
Diastolic BP	0.156	7.7 × 10^−2^	0.170	5.9 × 10^−2^
FPI	0.414 **	1.2 × 10^−4^	0.480 **	7.5 × 10^−6^
FPG	0.078	2.5 × 10^−1^	0.127	1.4 × 10^−1^
HOMA-IR	0.208 *	3.8 × 10^−2^	0.269 *	1.1 × 10^−2^
HbA1c	0.146	1.5 × 10^−1^	0.145	1.5 × 10^−1^
Total cholesterol	0.082	2.7 × 10^−1^	0.079	2.8 × 10^−1^
LDL	0.199	7.3 × 10^−2^	0.177	9.8 × 10^−2^
HDL	−0.295 *	1.4 × 10^−2^	−0.313 **	9.4 × 10^−3^
Triglycerides	0.097	2.4 × 10^−1^	0.176	9.9 × 10^−2^
Plasma Hcy	0.437 **	2.7 × 10^−3^	0.438 **	2.7 × 10^−3^
Plasma NO	−0.414 **	4.1 × 10^−5^	−0.423 **	2.7 × 10^−5^
Plasma Folate	−0.368 **	1.1 × 10^−3^	−0.353 **	1.6 × 10^−3^
Plasma Vitamin B12	−0.384 **	6.1 × 10^−4^	−0.393 **	4.6 × 10^−4^
Plasma Vitamin D	−0.622 **	5.9 × 10^−9^	−0.595 **	3.4 × 10^−8^
Alcohol consumption	0.516 **	2.2 × 10^−7^	0.457 **	5.6 × 10^−6^

Abbreviations: BMI, body mass index; VAT, visceral adipose tissues; BP, blood pressure; FPG, fasting plasma glucose; FPI, fasting plasma insulin; HOMA-IR, homeostatic model assessment for insulin resistance; HbA1c, glycosylated hemoglobin; HDL, high-density lipoprotein; LDL, low-density lipoprotein; Hcy, homocysteine; NO, nitric oxide. * for *p*-value < 0.05 and ** for *p*-value < 0.01.

**Table 4:** Summary of the genes that demonstrated significant differences in DNA methylation between obese and non-obese subjects.

Genes	Control	Obese	*p*-Value
	% of Methylated Fraction (Mean ± SE)		
ABCF1	72% ± 7%	63% ± 7%	2.3 × 10^−9^
BCL6	66% ± 5%	61% ± 1%	1.5 × 10^−4^
CCL25	54% ± 3%	52% ± 3%	1.3 × 10^−2^
CSF1	92% ± 17%	19% ± 9%	2.7 × 10^−32^
CXCL8	44% ± 3%	39% ±6%	4.1 × 10^−5^
CXCR2	67% ± 5%	63% ± 5%	1.2 × 10^−3^
FOXP1	72% ± 6%	78% ± 6%	1.9 × 10^−8^
GATA3	68% ± 6%	73% ± 6%	1.1 × 10^−4^
HDAC5	86% ± 5%	78% ± 5%	2.4 × 10^−16^
IGFBP3	71% ± 4%	65% ± 4%	1.6 × 10^−10^
IL12RB2	86% ± 5%	83% ± 5%	9.2 × 10^−6^
IL1R1	74% ± 4%	70% ± 4%	1.9 × 10^−5^
IL4	54% ± 4%	61% ± 9%	7.5 × 10^−5^
IL7	77% ± 5%	71% ± 4%	9.2 × 10^−10^
LTBP1	74% ± 4%	70% ± 5%	1.3 × 10^−5^
LTBR	84% ± 5%	79% ± 4%	1.2 × 10^−9^
MYD88	86% ± 12%	29% ± 8%	7.2 × 10^−21^
NFATC3	83% ± 8%	49% ± 16%	5.4 × 10^−20^
NFκB	84% ± 6%	80% ± 5%	3.2 × 10^−6^
PTGS1	82% ± 6%	79% ± 6%	5.1 × 10^−3^
RELB	72% ± 8%	68% ± 6%	5.8 × 10^−4^
S1PR2	73% ± 5%	67% ± 3%	1.4 × 10^−7^
SMAD3	77% ± 6%	70% ± 5%	2.6 × 10^−10^
TGFBR2	77% ± 1%	73% ± 1%	4.8 × 10^−23^
TLR2	32% ± 5%	26% ± 4%	6.7 × 10^−7^
TLR5	80% ±6%	33% ± 4%	1.7 × 10^−43^
TNFRSF8	50% ±5%	45% ± 3%	1.2 × 10^−5^
TRAF6	82% ±4%	77% ± 3%	2.0 × 10^−12^

ABCF1, ATP binding cassette subfamily F member 1; BCL6, B-cell lymphoma 6; CCL25, C-C motif chemokine ligand 25; CSF1, colony-stimulating factor 1; CXCL8, C-X-C motif chemokine ligand 8; CXCR2, C-X-C motif chemokine receptor 2; FOXP1, forkhead box P1; GATA3, GATA binding protein 3; HDAC5, histone deacetylase 5; IGFBP3, insulin-like growth factor binding protein 3; IL12RB2, interleukin 12 receptor subunit beta 2; IL1R1, interleukin 1 receptor type 1; IL; LTBP1, latent transforming growth factor-beta binding protein 1; LTBR, lymphotoxin beta receptor; MYD88, Myeloid differentiation primary response 88; NFATC3, nuclear factor of activated T cells 3; NFκB, nuclear factor kappa B; PTGS1, prostaglandin-endoperoxide synthase 1; RELB, v-rel reticuloendotheliosis viral oncogene homolog B; S1PR2, sphingosine-1-phosphate receptor 2; SMAD3, Mothers against decapentaplegic homolog 3; TGFBR2, transforming growth factor-beta receptor 2; TLR, toll-like receptor; TNFRSF8, TNF receptor superfamily member 8; TRAF6, TNF receptor-associated factor 6.

**Table 5:** Correlations between adipokine methylation score and cardiometabolic risk factors.

Variable	Adipokine Methylation Score	
	Correlation	Significance (2-Tailed)
Body weight	−0.797 **	5.07 × 10^−23^
Waist circumference	−0.717 **	2.04 × 10^−7^
BMI	−0.841 **	1.05 × 10^−27^
Total fat %	−0.776 **	8.09 × 10^−10^
Total lean %	0.767 **	1.52 × 10^−9^
VAT mass	−0.680 **	6.66 × 10^−5^
Heart rate	−0.259 **	4.94 × 10^−3^
Systolic BP	−0.380 **	5.60 × 10^−5^
Diastolic BP	−0.229 *	1.15 × 10^−2^
FPI	−0.570 **	2.04 × 10^−8^
FPG	−0.157	8.33 × 10^−2^
HOMA−IR	−0.331 **	1.45 × 10^−3^
HbA1c	−0.140	1.57 × 10^−1^
Total cholesterol	−0.039	3.85 × 10^−1^
LDL	−0.184	8.35 × 10^−2^
HDL	0.387 **	1.22 × 10^−3^
Triglycerides	−0.165	1.08 × 10^−1^
Plasma Hcy	−0.452 **	2.81 × 10^−3^
Plasma NO	0.459 **	1.62 × 10^−6^
Plasma Folate	0.375 **	4.92 × 10^−4^
Plasma Vitamin B12	0.459 **	1.75 × 10^−5^
Plasma Vitamin D	0.623 **	7.41 × 10^−10^
Brachial FMD	0.632 **	6.93 × 10^−12^
Arteriolar FID Δ60	0.952 **	1.25 × 10^−40^
Arteriolar NO	0.767 **	6.02 × 10^−9^
Alcohol consumption	−0.374 **	7.53 × 10^−5^
VAT vascularity	−0.875 **	1.29 × 10^−25^
VAT HIF1α	−0.854 **	2.65 × 10^−23^
VAT TET−1	−0.608 **	2.19 × 10^−9^
Plasma IL6	−0.482 **	1.69 × 10^−3^
Plasma IL1β	−0.501 **	3.31 × 10^−9^
Plasma TNFα	−0.434 **	1.79 × 10^−5^
Plasma IFNγ	−0.489 **	2.06 × 10^−7^
Plasma VEGF	−0.453 **	1.00 × 10^−5^
Plasma CRP	−0.710 **	1.48 × 10^−10^

*p*-values are from non-paired Student’s test (* *p* < 0.05). Abbreviations: BMI, body mass index; VAT, visceral adipose tissues; BP, blood pressure; FPG, fasting plasma glucose; FPI, fasting plasma insulin; HOMA-IR, homeostatic model assessment for insulin resistance; HbA1c, glycosylated hemoglobin; HDL, high-density lipoprotein; LDL, low-density lipoprotein; Hcy, homocysteine; NO, nitric oxide; FMD, flow-mediated dilation; FID, flow-induced dilation; HIF1α, hypoxia-inducible factor 1 alpha; TET1, Ten-eleven translocation methylcytosine dioxygenase 1; IL6, interleukin 6; IL1β, interleukin 1 beta; TNFα, tumor necrosis factor-alpha; IFNγ, interferon-gamma; VEGF, vascular endothelial growth factor; CRP, C-reactive protein. * for *p*-value < 0.05 and ** for *p*-value < 0.01.

**Table 6:** Correlations between vascular reactivity and cardiometabolic risk factors.

	Brachial Artery FMD		Arteriolar FID at Δ60	
	Pearson Correlation	Sig (2-Tailed)	Pearson Correlation	Sig (2-Tailed)
Body weight	−0.462 **	5.82 × 10^−7^	−0.873 **	6.23 × 10^−28^
Waist circumference	−0.332 *	1.69 × 10^−2^	−0.761 **	9.63 × 10^−9^
BMI	−0.518 **	1.42 × 10^−8^	−0.937 **	5.81 × 10^−40^
Total fat %	−0.350 **	7.45 × 10^−3^	−0.843 **	9.44 × 10^−14^
Total lean %	0.349 **	7.52 × 10^−3^	0.837 **	2.08 × 10^−13^
VAT mass	−0.236	1.09 × 10^−1^	−0.649 **	9.34 × 10^−5^
Heart rate	0.020	4.22 × 10^−1^	−0.187 *	4.34 × 10^−2^
Systolic BP	−0.232 **	9.68 × 10^−3^	−0.355 **	4.25 × 10^−4^
Diastolic BP	−0.105	1.49 × 10^−1^	−0.172	5.73 × 10^−2^
FPI	−0.304 **	2.11 × 10^−3^	−0.500 **	2.89 × 10^−6^
FPG	−0.098	1.83 × 10^−1^	−0.130	1.35 × 10^−1^
HOMA−IR	−0.190 *	3.93 × 10^−2^	−0.289 **	6.18 × 10^−3^
HbA1c	−0.136	1.50 × 10^−1^	−0.150	1.44 × 10^−1^
Total cholesterol	−0.139	1.35 × 10^−1^	0.003	4.91 × 10^−1^
LDL	−0.212 *	4.65 × 10^−2^	−0.139	1.56 × 10^−1^
HDL	0.155	1.09 × 10^−1^	0.373 **	2.33 × 10^−3^
Triglycerides	−0.006	4.83 × 10^−1^	−0.101	2.31 × 10^−1^
Plasma Hcy	−0.209	9.80 × 10^−2^	−0.420 **	3.89 × 10^−3^
Plasma NO	0.287 **	1.79 × 10^−3^	0.433 **	1.72 × 10^−5^
Plasma Folate	0.303 **	3.14 × 10^−3^	0.341 **	2.21 × 10^−3^
Plasma Vitamin B12	0.241 *	1.52 × 10^−2^	0.421 **	1.73 × 10^−4^
Plasma Vitamin D	0.367 **	3.16 × 10^−4^	0.633 **	2.70 × 10^−9^
Alcohol consumption	−0.239 **	8.00 × 10^−3^	−0.400 **	7.43 × 10^−5^
VAT HIF1α	−0.620 **	1.22 × 10^−10^	−0.895 **	4.36 × 10^−31^
VAT TET−1	−0.381 **	1.61 × 10^−4^	−0.653 **	6.49 × 10^−12^
Plasma IL6	−0.285 *	3.94 × 10^−2^	−0.453 **	2.13 × 10^−3^
Plasma CRP	−0.463 **	5.18 × 10^−5^	−0.798 **	2.43 × 10^−15^
Methylation Score	0.632 **	6.93 × 10^−12^	0.952 **	1.25 × 10^−40^

Abbreviations: BMI, body mass index; VAT, visceral adipose tissues; BP, blood pressure; FPG, fasting plasma glucose; FPI, fasting plasma insulin; HOMA-IR, homeostatic model assessment for insulin resistance; HbA1c, glycosylated hemoglobin; HDL, high-density lipoprotein; LDL, low-density lipoprotein; Hcy, homocysteine; NO, nitric oxide; HIF1α, hypoxia-inducible factor 1 alpha; TET1, Ten-eleven translocation methylcytosine dioxygenase 1; IL6, interleukin 6; CRP, C-reactive protein. * for *p*-value < 0.05 and ** for *p*-value < 0.01.

## Data Availability

The data presented in this study are available in the article and supplementary material.

## Supplementary Materials

The following are available online at https://www.mdpi.com/article/10.3390/biomedicines9081034/s1, Table S1: PCR primers, Table S2: PCR primers for ChIP-qPCR.

## References

[1] (2014). The Current State of Obesity Solutions in the United States: Workshop Summary.

[2] Khaodhiar L., McCowen K.C., Blackburn G.L. (1999). Obesity and its comorbid conditions. Clin. Cornerstone.

[3] Lian X., Gollasch M. (2016). A Clinical Perspective: Contribution of Dysfunctional Perivascular Adipose Tissue (PVAT) to Cardiovascular Risk. Curr. Hypertens. Rep..

[4] Trayhurn P. (2013). Hypoxia and Adipose Tissue Function and Dysfunction in Obesity. Physiol. Rev..

[5] Stafeev I.S., Michurina S.S., Podkuychenko N.V., Menshikov M.Y., Parfyonova Y.V., Vorotnikov A.V. (2019). Chemical Inducers of Obesity-Associated Metabolic Stress Activate Inflammation and Reduce Insulin Sensitivity in 3T3-L1 Adipocytes. Biochemistry (Moscow).

[6] Lawler H.M., Underkofler C.M., Kern P.A., Erickson C., Bredbeck B., Rasouli N. (2016). Adipose Tissue Hypoxia, Inflammation, and Fibrosis in Obese Insulin-Sensitive and Obese Insulin-Resistant Subjects. J. Clin. Endocrinol. Metab..

[7] Ye J. (2009). Emerging role of adipose tissue hypoxia in obesity and insulin resistance. Int. J. Obes. (Lond.).

[8] Nanduri J., Semenza G.L., Prabhakar N.R. (2017). Epigenetic changes by DNA methylation in chronic and intermittent hypoxia. Am. J. Physiol. Lung Cell. Mol. Physiol..

[9] Zhang S., Zhang Y., Jiang S., Liu Y., Huang L., Zhang T., Lu G., Gong K., Ji X., Shao G. (2014). The Effect of Hypoxia Preconditioning on DNA Methyltransferase and PP1gamm in Hippocampus of Hypoxia Preconditioned Mice. High Alt. Med. Biol..

[10] Hu X.-Q., Chen M., Dasgupta C., Xiao D., Huang X., Yang S., Zhang L. (2017). Chronic hypoxia upregulates DNA methyltransferase and represses large conductance Ca2+-activated K+ channel function in ovine uterine arteries. Biol. Reprod..

[11] Liu Q., Liu L., Zhao Y., Zhang J., Wang D., Chen J., He Y., Wu J., Zhang Z., Liu Z. (2011). Hypoxia Induces Genomic DNA Demethylation through the Activation of HIF-1alpha and Transcriptional Upregulation of MAT2A in Hepatoma Cells. Mol. Cancer Ther..

[12] Alivand M.R., Soheili Z.-S., Pornour M., Solali S., Sabouni F. (2017). Novel Epigenetic Controlling of Hypoxia Pathway Related to Overexpression and Promoter Hypomethylation of TET1 and TET2 in RPE Cells. J. Cell. Biochem..

[13] Wu S.C., Zhang Y. (2010). Active DNA demethylation: Many roads lead to Rome. Nat. Rev. Mol. Cell Biol..

[14] Ito S., D’Alessio A.C., Taranova O.V., Hong K., Sowers L.C., Zhang Y. (2010). Role of Tet proteins in 5mC to 5hmC conversion, ES-cell self-renewal and inner cell mass specification. Nature.

[15] Rasmussen K.D., Helin K. (2016). Role of TET enzymes in DNA methylation, development, and cancer. Genes Dev..

[16] Ali M.M., Phillips S.A., Mahmoud A.M. (2020). HIF1alpha/TET1 Pathway Mediates Hypoxia-Induced Adipocytokine Promoter Hypomethylation in Human Adipocytes. Cells.

[17] Haloul M., Vinjamuri S.J., Naquiallah D., Mirza M.I., Qureshi M., Hassan C., Masrur M., Bianco F.M., Frederick P., Cristoforo G.P. (2020). Hyperhomocysteinemia and Low Folate and Vitamin B12 Are Associated with Vascular Dysfunction and Impaired Nitric Oxide Sensitivity in Morbidly Obese Patients. Nutrients.

[18] Matthews D.R., Hosker J.P., Rudenski A.S., Naylor B.A., Treacher D.F., Turner R.C. (1985). Homeostasis model assessment: Insulin resistance and beta-cell function from fasting plasma glucose and insulin concentrations in man. Diabetologia.

[19] Mahmoud A.M., Szczurek M.R., Blackburn B.K., Mey J.T., Chen Z., Robinson A.T., Bian J.-T., Unterman T.G., Minshall R.D., Brown M.D. (2016). Hyperinsulinemia augments endothelin-1 protein expression and impairs vasodilation of human skeletal muscle arterioles. Physiol. Rep..

[20] Ali M.M., Naquiallah D., Hassan C., Masrur M., Bianco F.M., Frederick P., Cristoforo G., Gangemi A., Phillips S.A., Mahmoud A.M. (2020). Obesity-associated Hypoxia Contributes to Aberrant Methylation of Genes Implicated in Inflammation and vascular Function. FASEB J..

[21] Mahmoud A.M., Szczurek M., Hassan C., Masrur M., Gangemi A., Phillips S.A. (2019). Vitamin D Improves Nitric Oxide-Dependent Vasodilation in Adipose Tissue Arterioles from Bariatric Surgery Patients. Nutrients.

[22] Mahmoud A.M., Hwang C.-L., Szczurek M.R., Bian J.-T., Ranieri C., Gutterman D.D., Phillips S.A. (2019). Low-Fat Diet Designed for Weight Loss But Not Weight Maintenance Improves Nitric Oxide-Dependent Arteriolar Vasodilation in Obese Adults. Nutrients.

[23] Ali M.M., Naquiallah D., Qureshi M., Mirza M.I., Hassan C., Masrur M., Bianco F.M., Frederick P., Cristoforo G.P., Gangemi A. (2021). DNA methylation profile of genes involved in inflammation and autoimmunity correlates with vascular function in morbidly obese adults. Epigenetics.

[24] Mahmoud A.M., Solomon T.P.J., Phillips S.A., Kirwan J.P., Haus J.M. (2016). Aerobic Exercise Reduces NOX2 in Skeletal Muscle of Obese Insulin-resistant Adults Via Interfering with RAGE/p-IkB-α Axis. FASEB J..

[25] Livak K.J., Schmittgen T.D. (2001). Analysis of relative gene expression data using real-time quantitative PCR and the 2(-Delta Delta C(T)) Method. Methods.

[26] Selhub J. (2002). Folate, vitamin B12 and vitamin B6 and one carbon metabolism. J. Nutr. Health Aging.

[27] Wierzbicki A.S. (2007). Homocysteine and cardiovascular disease: A review of the evidence. Diabetes Vasc. Dis. Res..

[28] Humphrey L.L., Fu R., Rogers K., Freeman M., Helfand M. (2008). Homocysteine Level and Coronary Heart Disease Incidence: A Systematic Review and Meta-analysis. Mayo Clin. Proc..

[29] Gibson A., Woodside J.V., Young I.S., Sharpe P.C., Mercer C., Patterson C.C., McKinley M.C., Kluijtmans L.A., Whitehead A.S., Evans A. (2008). Alcohol increases homocysteine and reduces B vitamin concentration in healthy male volunteers—A randomized, crossover intervention study. QJM Int. J. Med..

[30] Alegría-Torres J.A., Baccarelli A., Bollati V. (2011). Epigenetics and lifestyle. Epigenomics.

[31] Zhang X., Ho S.-M. (2011). Epigenetics meets endocrinology. J. Mol. Endocrinol..

[32] Stoger R. (2008). Epigenetics and obesity. Pharmacogenomics.

[33] Ziegler A.K., Damgaard A., Mackey A.L., Schjerling P., Magnusson P., Olesen A.T., Kjaer M., Scheele C. (2019). An anti-inflammatory phenotype in visceral adipose tissue of old lean mice, augmented by exercise. Sci. Rep..

[34] Balamurugan K. (2016). HIF-1 at the crossroads of hypoxia, inflammation, and cancer. Int. J. Cancer.

[35] Landecho M.F., Tuero C., Valentí V., Bilbao I., De La Higuera M., Fruhbeck G. (2019). Relevance of Leptin and Other Adipokines in Obesity-Associated Cardiovascular Risk. Nutrients.

[36] Bullo M., Casas-Agustench P., Amigo-Correig P., Aranceta J., Salas-Salvado J. (2007). Inflammation, obesity and comorbidities: The role of diet. Public Health Nutr..

[37] Unamuno X., Gomez-Ambrosi J., Rodriguez A., Becerril S., Fruhbeck G., Catalan V. (2018). Adipokine dysregulation and adipose tissue inflammation in human obesity. Eur. J. Clin. Investig..

[38] Fujisaka S., Usui I., Ikutani M., Aminuddin A., Takikawa A., Tsuneyama K., Mahmood A., Goda N., Nagai Y., Takatsu K. (2013). Adipose tissue hypoxia induces inflammatory M1 polarity of macrophages in an HIF-1alpha-dependent and HIF-1alpha-independent manner in obese mice. Diabetologia.

[39] Lin Q., Yun Z. (2015). The Hypoxia-Inducible Factor Pathway in Adipocytes: The Role of HIF-2 in Adipose Inflammation and Hypertrophic Cardiomyopathy. Front. Endocrinol. (Lausanne).

[40] Lin G., Sun W., Yang Z., Guo J., Liu H., Liang J. (2017). Hypoxia induces the expression of TET enzymes in HepG2 cells. Oncol. Lett..

[41] Wu M.-Z., Chen S.-F., Nieh S., Benner C., Ger L.-P., Jan C.-I., Ma L., Chen C.-H., Hishida T., Chang H.-T. (2015). Hypoxia Drives Breast Tumor Malignancy through a TET–TNFalpha-–p38–MAPK Signaling Axis. Cancer Res..

[42] Shahrzad S., Bertrand K., Minhas K., Coomber B.L. (2007). Induction of DNA Hypomethylation by Tumor Hypoxia. Epigenetics.

[43] Tsai Y.-P., Chen H.-F., Chen S.-Y., Cheng W.-C., Wang H.-W., Shen Z.-J., Song C., Teng S.-C., He C., Wu K.-J. (2014). TET1 regulates hypoxia-induced epithelial-mesenchymal transition by acting as a co-activator. Genome Biol..

[44] De Luca C., Olefsky J.M. (2008). Inflammation and insulin resistance. FEBS Lett..

[45] Judd S.E., Tangpricha V. (2009). Vitamin D Deficiency and Risk for Cardiovascular Disease. Am. J. Med Sci..

[46] Wahl S., Drong A., Lehne B., Loh M., Scott W.R., Kunze S., Tsai P.-C., Ried J.S., Zhang W., Yang Y. (2017). Epigenome-wide association study of body mass index, and the adverse outcomes of adiposity. Nature.

[47] Campanella G., Gunter M.J., Polidoro S., Krogh V., Palli D., Panico S., Sacerdote C., Tumino R., Fiorito G., Guarrera S. (2018). Epigenome-wide association study of adiposity and future risk of obesity-related diseases. Int. J. Obes. (Lond.).

[48] Sayols-Baixeras S., Subirana I., Fernández-Sanlés A., Senti M., Lluís-Ganella C., Marrugat J., Elosua R. (2017). DNA methylation and obesity traits: An epigenome-wide association study. The REGICOR study. Epigenetics.

[49] Fradin D., Boelle P.-Y., Belot M.-P., Lachaux F., Tost J., Besse C., Deleuze J.-F., De Filippo G., Bougneres P. (2017). Genome-Wide Methylation Analysis Identifies Specific Epigenetic Marks In Severely Obese Children. Sci. Rep..

[50] Ding X., Zheng D., Fan C., Liu Z., Dong H., Lu Y., Qi K. (2015). Genome-wide screen of DNA methylation identifies novel markers in childhood obesity. Gene.

[51] Huang R.C., Garratt E.S., Pan H., Wu Y., Davis E.A., Barton S.J., Burdge G.C., Godfrey K.M., Holbrook J.D., Lillycrop K.A. (2015). Genome-wide methylation analysis identifies differentially methylated CpG loci associated with severe obesity in childhood. Epigenetics.

[52] Castellano-Castillo D., Moreno-Indias I., Sanchez-Alcoholado L., Ramos-Molina B., Alcaide-Torres J., Morcillo S., Ocana-Wilhelmi L., Tinahones F., Queipo-Ortuno M.I., Cardona F. (2019). Altered Adipose Tissue DNA Methylation Status in Metabolic Syndrome: Relationships Between Global DNA Methylation and Specific Methylation at Adipogenic, Lipid Metabolism and Inflammatory Candidate Genes and Metabolic Variables. J. Clin. Med..

[53] Dunstan J., Bressler J.P., Moran T.H., Pollak J.S., Hirsch A.G., Bailey-Davis L., Glass T.A., Schwartz B.S. (2017). Associations of LEP, CRH, ICAM-1, and LINE-1 methylation, measured in saliva, with waist circumference, body mass index, and percent body fat in mid-childhood. Clin. Epigenet..

[54] Kim A.Y., Park Y.J., Pan X., Shin K.C., Kwak S.-H., Bassas A.F., Sallam R.M., Park K.S., Alfadda A.A., Xu A. (2015). Obesity-induced DNA hypermethylation of the adiponectin gene mediates insulin resistance. Nat. Commun..

[55] Zhang X., Gu H.F., Frystyk J., Efendic S., Brismar K., Thorell A. (2019). Analyses of IGFBP2 DNA methylation and mRNA expression in visceral and subcutaneous adipose tissues of obese subjects. Growth Horm. IGF Res..

[56] Wei L., Liu S., Su Z., Cheng R., Bai X., Li X. (2014). LINE-1 Hypomethylation is Associated with the Risk of Coronary Heart Disease in Chinese Population. Arq. Bras. Cardiol..

[57] Lin R.-T., Hsi E., Lin H.-F., Liao Y.-C., Wang Y.-S., Juo S.H. (2014). LINE-1 Methylation is Associated with an Increased Risk of Ischemic Stroke in Men. Curr. Neurovasc. Res..

[58] Baccarelli A., Wright R., Bollati V., Litonjua A., Zanobetti A., Tarantini L., Sparrow D., Vokonas P., Schwartz J. (2010). Ischemic Heart Disease and Stroke in Relation to Blood DNA Methylation. Epidemiology.

[59] Poli V.F.S., Sanches R.B., Moraes A.D.S., Fidalgo J.P.N., Nascimento M.A., Bresciani P., Andrade-Silva S.G., Cipullo M.A.T., Clemente J.C., Caranti D.A. (2017). The excessive caloric intake and micronutrient deficiencies related to obesity after a long-term interdisciplinary therapy. Nutrition.

[60] Kim Y.I. (2004). Folate and DNA methylation: A mechanistic link between folate deficiency and colorectal cancer?. Cancer Epidemiol. Biomark. Prev..

[61] Kulis M., Esteller M. (2010). DNA Methylation and Cancer. Adv. Genet..

[62] Wang X., Guan Z., Chen Y., Dong Y., Niu Y., Wang J., Zhang T., Niu B. (2015). Genomic DNA Hypomethylation Is Associated with Neural Tube Defects Induced by Methotrexate Inhibition of Folate Metabolism. PLoS ONE.

