# Supplementary material for: Adipose Tissue Hypoxia Correlates with Adipokine Hypomethylation and Vascular Dysfunction
Source: Biomedicines. 2021 Aug 18;9(8):1034. doi: 10.3390/biomedicines9081034 (PMC8394952; doi:10.3390/biomedicines9081034)
Supplement: Supplementary file 1 [file biomedicines-09-01034-s001.zip › biomedicines-1329629-supplementary.pdf]

Supplementary Table S1. PCR primers

Gene	Fw primers	Rv primers	Tm	product size
**ABCF1**	CGGAAAAACCAAGATGAGGA	GTCGATGCCAAAATCCAAGT	60	186
**BCL6**	GATGAGATTGCCCTGCATTT	TTCTTCCAGTTGCAGGCTTT	60	203
**CCL25**	AAGGTTTTTGCAAAGCTCCA	TGTAGGGCGACGGTTTTATC	60	239
**CSF1**	CCCAGTGTCATCCTGGTCTT	GAGACTGTTCTGTGCGTCCA	60	193
**CXCL8**	TAGCAAAATTGAGGCCAAGG	AAACCAAGGCACAGTGGAAC	60	227
**CXCR2**	ACAGCTACTTGGGAGGCTGA	TGCAGTGGTCACACCATTTT	60	193
**FOXP1**	TTCAGGGGTAAGACGTGACC	AGACCGCCGCACTCTAGTAA	60	173
**GATA3**	CTCATTAAGCCCAAGCGAAG	TTTTTCGGTTTCTGGTCTGG	60	205
**HDAC5**	CATCTCTGCAGACCCCTCTC	CCCACACACTTTCACCCTCT	60	163
**IGFBP3**	CAGAGACTCGAGCACAGCAC	GATGACCGGGGTTTAAAGGT	60	194
**IL12RB2**	ACGGAGTCGACCCTACAATG	TCCCCTTTTCCTCTGTGATG	60	185
**IL1R1**	TATGCCTCATGCTGACTTGC	CTCTGGTGATCCACCCACTT	60	232
**IL4**	GCCACCATGAGAAGGACACT	ACTCTGGTTGGCTTCCTTCA	60	152
**IL7**	CCAAGGCGTTGAGAGATCAT	ATCCGCCAGCAGTGTACTTT	60	236
**LTBP1**	CTTCCGCTGCCTCTGTTATC	CATGGGGCTGTACTCTTGGT	60	172
**LTBR**	CTCATCCCCAAGCTTCAGAG	GAATCCTACCCAACCCCCTA	60	158
**MYD88**	GCACATGGGCACATACAGAC	GACATGGTTAGGCTCCCTCA	60	159
**NFATC3**	GCAATCTCCTTGCCACTCTC	ACCAGGTGAAGGAACAGGTG	60	181
**NFκB**	CCTGGATGACTCTTGGGAAA	TCAGCCAGCTGTTTCATGTC	60	174
**PTGS1**	GAGTACTGGAAGCCGAGCAC	GCACTCTGGAATGACAAGCA	60	234
**RELB**	TCCCAACCAGGATGTCTAGC	AGCCATGTCCCTTTTCCTCT	60	160
**S1PR2**	CACCTGGCGGTACAAAGAAT	GTCAAGTGGCAGCTGATGAA	60	210
**SMAD3**	TGCTGGTGACTGGATAGCAG	CTCCTTGGAAGGTGCTGAAG	60	176
**TGFBR2**	CCATGTCTCACAGCCAGCTA	CCAGGAGAAATAAGGGCACA	60	155
**TLR2**	TCCCAAAGCATGCTACTCCT	CTTCCTTGGAGAGGCTGATG	60	220
**TLR5**	AAAAATGGGATGGTCCATGA	TGGAGAAGCCGAAGGTAAGA	60	237
**TNFRSF8**	GAGGCTCCAGCATCTAGTGG	GCCTGGTGGTTAAGGTCTGA	60	248
**TRAF6**	CGCCTGAGAATCACTGCATA	ATAAGCAAGCGCAAAGGAAA	60	199

Supplementary Table S2. PCR primers for ChIP-qPCR

	Promoter ID (EPD)	Fw primers	Rv primers	Tm	Product size
**ABCF1**	FP009360	GCGGTGGCAGAACTAGAAAC	CTTGCTCCAGTCGCGTAAAC	60.0	216
**BCL6**	FP006453	AATCCGAGACGCTCTGCTTA	GCTTCGGACTTTACCACTGC	60.0	165
**CCL25**	FP024918	AGGTGGAGAGGAGGAGGAAG	ACCGATATCCACTGGGTCAA	60.0	156
**CSF1**	FP001431	CAAAGGATTTCCCTCCCTTC	CTTCCAAGCCTTCAGCAAAC	60.0	227
**CXCL8**	FP007017	CATCAGTTGCAAATCGTGGA	GTTCCTTCCGGTGGTTTCTT	60.0	179
**CXCR2**	FP004554	AAGGAAGCCAGTTGCAGTGT	ACTGCCTCTCTGGTCCTTGA	60.0	189
**FOXP1**	FP005654	CTGCGTGAATGAGCTGTGTT	ATCAGTTGGAGCTTGCAGGT	60.0	165
**GATA3**	FP014224	CACATTTAAAGGGCCAGAGC	AAGGAAACTGCAACCCAAAC	60.0	200
**HDAC5**	FP023363	CCCTTTAAGGAGCTGTCACG	ACATTCGGAGACGTCACTCC	60.0	179
**IGFBP3**	FP011019	CACGAGGTACACACGAATGC	CGGGTCACCTTGTCGTCTAC	60.5	226
**IL12RB2**	FP001087	TCAACAAAGTGCCACGTCTC	TCTCTCCACCGTCAGTTTCC	60.2	169
**IL1R1**	FP003766	TTAGTGCACAAAGCCAGTCG	GGCGGCTCCACTAGAGTTAG	60.1	199
**IL4**	FP008437	TGTGGCCTCTCCCTTCTATG	GCCAATCAGCACCTCTCTTC	60.2	213
**IL7**	FP012538	ACGTCCTGCGTTTAATTTGG	ACGATGCCAAGTCGTCTTTT	60.0	215
**LTBP1**	FP003199	CTCTTTGTCTGCCCGTGAAT	CAACCCGACAGGTTTAAGGA	60.2	194
**LTBR**	FP017175	CCACAGTAGGGCAGGACAAC	AGGGAGAGCAGAGGGAGTTC	60.0	194
**MYD88**	FP005150	GACCGTATGAACCCCTCAGA	GGGGGCGTAAGAAGTAGGAA	60.4	203
**NFATC3**	FP022060	CGTGACTTCCTTCCTTCTCG	GTGCCTAAGCCTCCACTGTC	60.0	235
**NFκB**	FP007239	TGGACCGCATGACTCTATCA	GGCTCTGGCTTCCTAGCAG	60.2	158
**PTGS1**	FP013782	CATCCCCAGTTCTGTCCCTA	TAGTGTATGCTCGCCGCTAA	60.0	157
**RELB**	FP025932	GGTGTAGCGAGACCTTGGAG	ATCACGCCTTACCCATTGAG	60.0	181
**S1PR2**	FP001359	TGCGTCTCAGCAGTTCAGAT	CGACTGACTGCGTAGTGCTC	60.0	240
**SMAD3**	FP020691	GGTGACAGCACTTGGAAAGG	GCTAGCTTTGATGCGCAGAT	60.7	191
**TGFBR2**	FP005101	AAAGAAATGTGCTCGCGACT	AGGAGTTCCTCGCTCCAAGT	60.3	225
**TLR2**	FP013751	CGCTTTCACTTCCTCTCACC	ATCATCCTGGCATCATCCTC	60.0	183
**TLR5**	FP002699	GAACATGGGATGGTGCTTCT	GCAGCTCAGGGTTTTCTCTG	60.0	224
**TNFRSF8**	FP000233	CTTGGGGCCTCCTAATATCC	CGTGCAGAGATGACTCAGGA	60.0	198
**TRAF6**	FP015788	TCCTGAAGGTCAAGCGATCT	GTCAATGGGTGTGTGTTGGA	60.2	166
